# Thoracic Segmental Spinal Anesthesia for Bullectomy in a Patient With Severe Pulmonary Compromise: A Case Report

**DOI:** 10.1155/cria/2418831

**Published:** 2026-07-19

**Authors:** Sari Anani, Seedra Nassr, Tasnim Herbawi, Mamoun Halawa, Majde Ghanem, Fayez Mujahed

**Affiliations:** ^1^ Faculty of Medicine, Al-Quds University, Jerusalem, State of Palestine, alquds.edu; ^2^ Al-Ahli Hospital, Hebron, State of Palestine, ahlihospital.com

## Abstract

**Background:**

Thoracic surgery is conventionally performed under general anesthesia with one‐lung ventilation. However, this approach may pose considerable perioperative risks in selected patients with advanced pulmonary disease and significant medical comorbidities. Thoracic segmental spinal anesthesia (TSSA), combined with adjunctive regional techniques, has emerged as a potential alternative in carefully selected high‐risk patients undergoing nonintubated thoracic surgery [1–3].

**Presentation:**

A 76‐year‐old man with spontaneous left‐sided pneumothorax, persistent air leak despite chest tube drainage and two autologous blood patch pleurodesis attempts, resolving pneumonia, cerebellar atrophy, parkinsonian tremor, and anterior fascicular block was referred for surgical management. His respiratory reserve was severely compromised, with oxygen saturation decreasing to 72% before admission and persistent oxygen dependence despite chest drainage [1,2]. Pulmonary function testing could not be performed because of his clinical condition. Following multidisciplinary discussion, conventional general anesthesia with one‐lung ventilation was considered to carry a substantial perioperative respiratory risk. The patient subsequently underwent awake video‐assisted thoracoscopic surgery (VATS) under low‐dose TSSA at the T5–T6 interspace using 6 mg isobaric bupivacaine, combined with an ultrasound‐guided cervical vagus nerve block using 4 mL of 1% lidocaine to suppress the intraoperative cough reflex. The patient remained awake, maintained spontaneous ventilation throughout surgery, demonstrated stable hemodynamics, and required no conversion to general anesthesia. Wedge resection of a giant pulmonary bulla and mechanical pleurodesis were completed successfully without intraoperative complications. Postoperatively, lung re‐expansion was achieved, the persistent air leak resolved, and the patient recovered without neurological complications, postdural puncture headache, urinary retention, dysphagia, hoarseness, or respiratory deterioration. He was discharged home on postoperative day two in stable condition.

**Conclusion:**

This case illustrates that TSSA combined with ultrasound‐guided vagus nerve block may represent a feasible anesthetic option for carefully selected high‐risk patients undergoing awake thoracoscopic surgery when conventional general anesthesia is considered undesirable. Larger prospective studies are required to further evaluate the safety, efficacy, and patient selection criteria for this approach [2,3].

## 1. Introduction

Thoracic surgery is traditionally performed under general anesthesia using endotracheal intubation and one‐lung ventilation (OLV) to facilitate surgical exposure [1]. Although this remains the standard anesthetic approach, airway manipulation, positive‐pressure ventilation, and neuromuscular blockade may increase perioperative morbidity in selected patients with severe pulmonary dysfunction, advanced age, or significant medical comorbidities [1, 2].

Over the last decade, increasing interest has developed in nonintubated thoracic surgery (NITS) as a strategy to preserve spontaneous ventilation while avoiding complications associated with general anesthesia. Several regional anesthetic techniques have been described for awake thoracic surgery, including thoracic epidural anesthesia, paravertebral block, intercostal nerve blocks, and thoracic segmental spinal anesthesia (TSSA). Among these techniques, TSSA offers rapid onset, dense segmental anesthesia, limited local anesthetic requirements, and potentially less extensive sympathetic blockade when low‐dose local anesthetics are used [2, 3].

Despite these potential advantages, thoracic spinal anesthesia remains relatively uncommon because of concerns regarding inadvertent spinal cord injury. Recent anatomical and clinical studies, however, suggest that thoracic spinal anesthesia can be performed safely by experienced anesthesiologists using meticulous technique, careful patient selection, and low‐dose local anesthetic administration.

Suppression of the intraoperative cough reflex represents another major challenge during awake thoracic surgery. Ultrasound‐guided vagus nerve block has recently gained attention as an adjunctive technique capable of reducing cough while preserving spontaneous ventilation and avoiding systemic sedative medications [4, 5].

Here, we report a CARE‐compliant case of successful awake video‐assisted thoracoscopic bullectomy and mechanical pleurodesis performed under TSSA combined with ultrasound‐guided vagus nerve block in a patient with severe pulmonary compromise who was considered to be at high risk for conventional general anesthesia.

## 2. Case Presentation

### 2.1. Patient Information

A 76‐year‐old male presented with progressively worsening respiratory symptoms over approximately one month. Initially, he developed an intermittent productive cough associated with low‐grade fever and was diagnosed with community‐acquired pneumonia, for which he completed a course of antibiotic therapy. Although the infectious symptoms gradually improved, he experienced progressive hypoxemia despite treatment.

Home pulse oximetry documented a gradual decline in oxygen saturation to 88%, followed by sudden‐onset dyspnea with oxygen saturation falling to 72%, prompting emergency presentation. Needle decompression was initially performed, followed by insertion of a left‐sided chest tube after transfer to a tertiary referral center.

### 2.2. Clinical Findings

**FIGURE 1:**
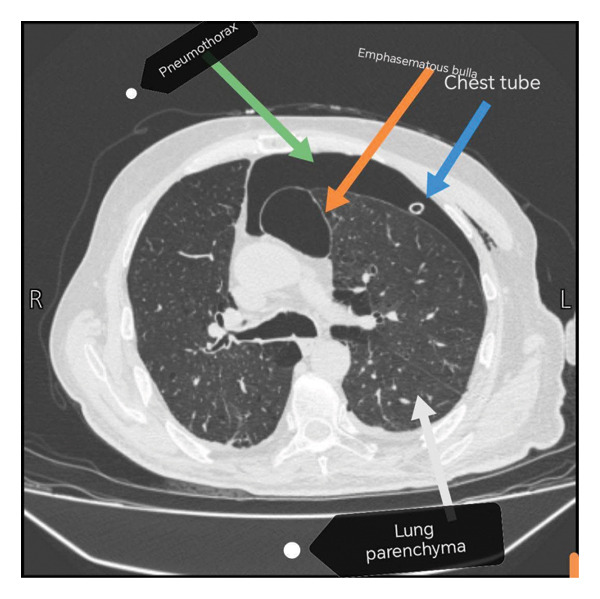
Chest CT scan showing the green arrow: pneumothorax, orange arrow: emphysematous bulla, blue arrow: chest tube; and white arrow: lung parenchyma. Preoperative computed tomography demonstrating left‐sided pneumothorax with appropriately positioned thoracostomy tube.

**FIGURE 2:**
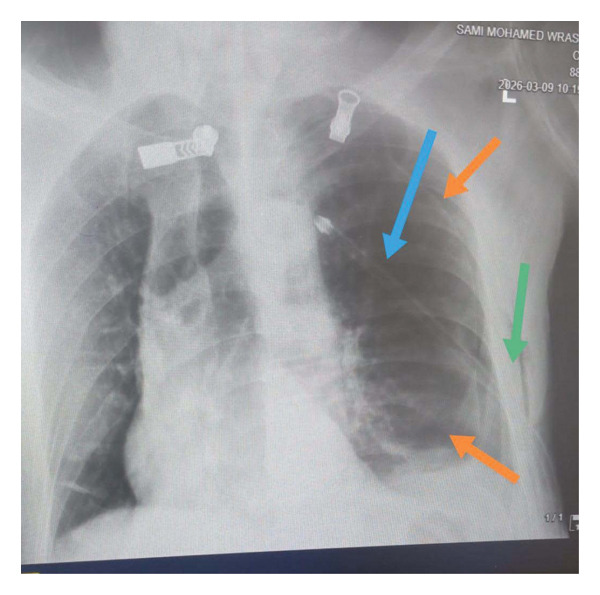
CXR showing orange arrow: lung border; blue arrow: chest tube in place; green arrow: subcutaneous emphysema. Computed tomography demonstrating extensive left‐sided subcutaneous emphysema surrounding the thoracostomy tube.

Computed tomography (CT) demonstrated a persistent left spontaneous pneumothorax despite an appropriately positioned left thoracostomy tube, accompanied by mediastinal shift (Figure 1). The CT scan also demonstrated extensive left‐sided subcutaneous emphysema surrounding the thoracostomy tube (Figure 2). Imaging also demonstrated radiological evidence of resolving pneumonia without laboratory evidence of ongoing systemic infection.

Past medical history was significant for:

1. Cerebellar atrophy
2. Parkinsonian tremor
3. Previous orthopedic surgery
4. Anterior fascicular block

On examination, breath sounds were markedly reduced over the left hemithorax. Blood pressure remained between 106/60 and 140/70 mmHg throughout admission. Oxygen supplementation remained necessary despite chest tube drainage.

### 2.3. Diagnostic Assessment

Initial laboratory investigations showed preserved renal function, normal coagulation profile, and stable hematologic parameters suitable for surgery.

Hemoglobin ranged from 13.65 to 15.10 g/dL, platelet count from 224–303 × 10^3^/μL, and white blood cell count between 4.7 and 10.7 × 10^3^/μL. Serum creatinine remained stable (0.68–0.73 mg/dL), electrolytes were within normal limits, and coagulation studies showed an INR of 1.19–1.24 with normal activated partial thromboplastin time. C‐Reactive protein remained only mildly elevated and subsequently normalized, supporting resolution rather than active pneumonia.

**FIGURE 3:**
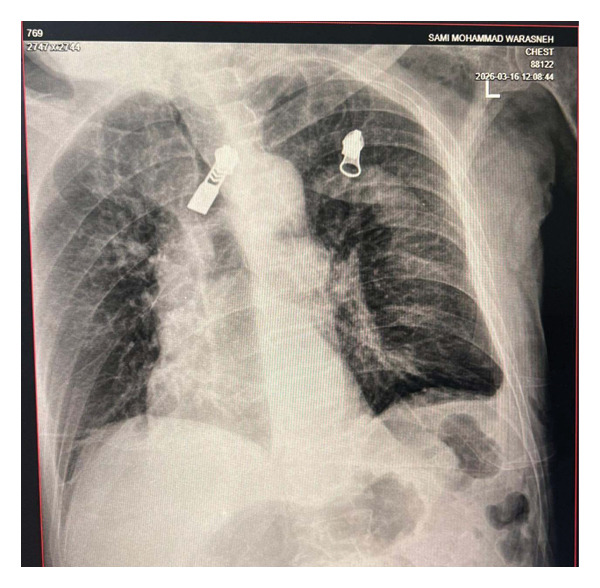
CXR showing left moderate pneumothorax despite left chest tube. The pneumothorax is causing a mass effect on mediastinal structure with a mediastinal shift to the right—diffuse emphysematous changes in the lungs—left upper lobe pulmonary bulla measures 5.5 × 4 cm. Preoperative computed tomography showing a 5.5 × 4 cm pulmonary bulla with persistent pneumothorax and rightward mediastinal shift.

Preoperative imaging additionally demonstrated a large pulmonary bulla measuring approximately 5.5 × 4 cm associated with persistent pneumothorax and rightward mediastinal shift (Figure 3). Two consecutive autologous blood patch pleurodesis procedures resulted in partial reduction of the air leak but failed to achieve complete lung expansion.

Pulmonary function testing and formal cardiopulmonary exercise assessment could not be performed because of the patient’s respiratory condition and persistent pneumothorax.

Following multidisciplinary evaluation involving thoracic surgery, pulmonology, and anesthesiology teams, the patient was considered to be at high perioperative risk. The patient was classified as American Society of Anesthesiologists (ASA) Physical Status IV because of conventional general anesthesia owing to the combination of:

1. Advanced age,
2. Persistent spontaneous pneumothorax with prolonged air leak,
3. Ongoing oxygen requirement,
4. Resolving recent pneumonia,
5. Inability to perform pulmonary function testing,
6. Underlying cerebellar atrophy and parkinsonian tremor,
7. Anticipated difficulty tolerating OLV,
8. Increased risk of postoperative respiratory failure and prolonged mechanical ventilation.

After discussion of the available anesthetic options with the patient and his family, written informed consent was obtained for awake video‐assisted thoracoscopic surgery (VATS) under TSSA combined with ultrasound‐guided vagus nerve block.

### 2.4. Therapeutic Intervention

After multidisciplinary discussion involving the thoracic surgery, pulmonology, and anesthesiology teams, the patient was scheduled for awake VATS with wedge resection of the pulmonary bulla and mechanical pleurodesis. Although he remained hemodynamically stable after chest tube drainage, he continued to have a persistent air leak requiring continuous suction, persistent oxygen dependence, and significant pulmonary compromise despite conservative management and two autologous blood patch pleurodesis attempts. In addition, his advanced age, resolving recent pneumonia, cerebellar atrophy with parkinsonian tremor, inability to undergo pulmonary function testing, and the anticipated need for OLV were considered to substantially increase the perioperative risk associated with conventional general anesthesia. Following careful multidisciplinary evaluation and detailed discussion with the patient and his family regarding the potential benefits and risks of the proposed anesthetic technique, written informed consent was obtained.

Upon arrival in the operating room, standard ASA monitoring was applied, including continuous electrocardiography, pulse oximetry, respiratory rate monitoring, and noninvasive blood pressure measurements at regular intervals. Supplemental oxygen was administered via nasal cannula throughout the procedure to maintain oxygen saturation above 94%. Intravenous access was secured before the block, and vasopressors, advanced airway equipment, and a complete setup for immediate conversion to general anesthesia with double‐lumen endotracheal intubation were prepared and readily available throughout the operation should respiratory or surgical circumstances require conversion.

The patient was initially positioned sitting upright, which allowed optimal identification of thoracic anatomical landmarks while minimizing movement related to his mild parkinsonian tremor. Following sterile preparation of the thoracic region using chlorhexidine antiseptic solution and sterile draping, the skin and subcutaneous tissue over the intended puncture site were infiltrated with 2 mL of 1% lidocaine. The T5–T6 intervertebral space was identified using surface anatomical landmarks. This level was deliberately selected because it provides adequate sensory blockade for thoracoscopic procedures involving the fourth and fifth intercostal spaces while minimizing cephalad spread of local anesthetic and reducing the likelihood of extensive sympathetic blockade.

A 25‐gauge pencil‐point spinal needle was introduced through a midline approach. Cerebrospinal fluid was obtained on the first attempt, confirming correct intrathecal placement. No paresthesia, pain, blood aspiration, or patient discomfort occurred during needle advancement or injection. Six milligrams of preservative‐free isobaric 0.5% bupivacaine were then injected slowly into the thoracic subarachnoid space without the addition of intrathecal opioids or other adjuvants. The procedure was performed by an anesthesiologist experienced in thoracic regional anesthesia. Following the spinal injection, the patient was carefully repositioned into the lateral decubitus position. Sensory blockade was assessed using loss of cold sensation and pinprick testing before skin incision, demonstrating an effective block extending approximately from the T3 to T8 dermatomes, providing complete surgical anesthesia while preserving lower‐extremity motor function and limiting sympathetic blockade. Throughout block placement, the patient’s hemodynamic parameters remained stable without clinically significant hypotension or bradycardia, and no vasopressor support was required.

Because suppression of the cough reflex was essential during manipulation of the lung and visceral pleura, an ultrasound‐guided ipsilateral cervical vagus nerve block was subsequently performed. A high‐frequency linear ultrasound transducer was placed transversely over the neck at the level of the thyroid cartilage. The common carotid artery and internal jugular vein were first identified, and the vagus nerve was visualized within the carotid sheath between these two vascular structures. Under continuous real‐time ultrasound guidance, a fine regional anesthesia needle was advanced using an in‐plane lateral‐to‐medial approach, allowing visualization of the needle tip throughout the procedure. After careful negative aspiration to exclude intravascular placement, 4 mL of 1% lidocaine was injected incrementally around the vagus nerve until circumferential spread of local anesthetic surrounding the nerve was confirmed sonographically. The purpose of the block was to suppress the intraoperative cough reflex while preserving spontaneous ventilation and avoiding the need for systemic anesthetic agents or airway instrumentation. Potential complications including transient hoarseness, recurrent laryngeal nerve blockade, dysphagia, vocal cord paresis, airway compromise, local anesthetic systemic toxicity, hematoma, and infection were anticipated and carefully monitored for throughout the perioperative period. None of these complications occurred. The patient maintained normal phonation postoperatively, tolerated oral intake without dysphagia, and exhibited no evidence of airway compromise or neurological deficits attributable to the vagus nerve block.

**FIGURE 4:**
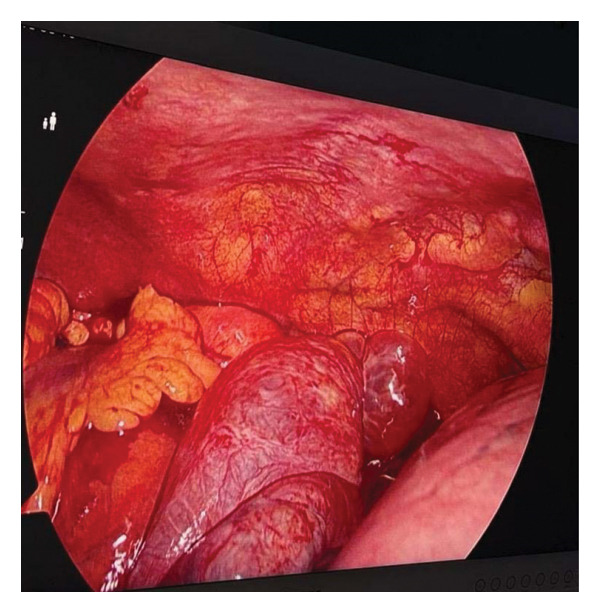
Thoracoscopic view demonstrating a large bulla. Intraoperative thoracoscopic view demonstrating the giant pulmonary bulla before wedge resection.

**FIGURE 5:**
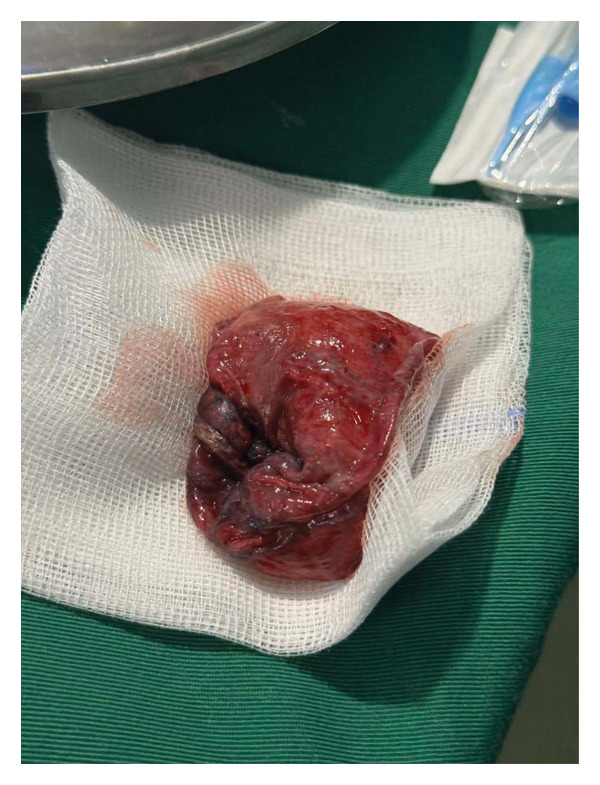
Resected bulla specimen. Gross specimen of the resected pulmonary bulla following wedge resection.

Following confirmation of an adequate sensory block, awake VATS was commenced without general anesthesia or endotracheal intubation. The patient remained fully awake, cooperative, breathing spontaneously, and comfortable throughout the entire procedure, including stapling of the lung parenchyma and mechanical pleurodesis. A 4‐cm utility incision was made in the fourth intercostal space anterior to the anterior axillary line. Thoracoscopic exploration demonstrated mild apical pleural adhesions and a large pulmonary bulla arising from the superior segment of the left lower lobe without additional pleural abnormalities (Figure 4). Wedge resection of the bulla was performed using a medium‐thickness purple endoscopic linear stapling cartridge, and the resected specimen was removed intact for pathological examination (Figure 5) followed by mechanical pleurodesis involving nearly the entire parietal pleural surface. A 20‐Fr chest tube was inserted under direct thoracoscopic vision. At the conclusion of the procedure, the lung was gradually re‐expanded using continuous positive airway pressure (CPAP) delivered through a well‐fitted face mask while the patient continued spontaneous ventilation, thereby avoiding endotracheal intubation and mechanical ventilation. The thoracoscopic incisions were then closed in the standard fashion.

Throughout the operation, the patient remained awake, communicative, and hemodynamically stable, with no episodes of clinically significant hypotension, bradycardia, hypoxemia, arrhythmia, excessive bleeding, or respiratory deterioration. The vagus nerve block provided effective suppression of the cough reflex, allowing uninterrupted thoracoscopic manipulation and completion of the wedge resection and pleurodesis without conversion to general anesthesia. Estimated blood loss was minimal, and no intraoperative complications occurred.

**FIGURE 6:**
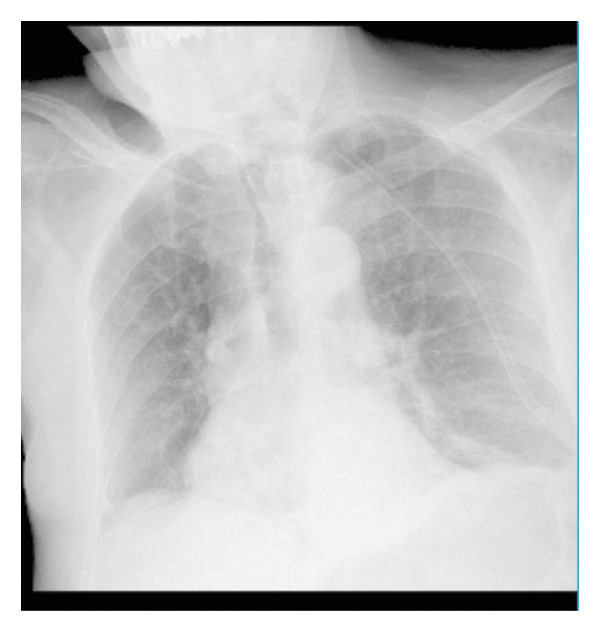
Immediate post‐op CXR shows lung expansion with no residual pneumothorax. Postoperative chest radiograph demonstrating complete left lung re‐expansion without residual pneumothorax.

Following surgery, the patient was transferred to the surgical intensive care unit for routine observation. Postoperative analgesia consisted of a multimodal opioid‐sparing regimen using intravenous paracetamol (1 g every 8 h) and metamizole 500 mg twice daily, providing satisfactory pain control without respiratory depression. Oxygen therapy was continued via nasal cannula at 3 L/min, maintaining oxygen saturation at approximately 94%. The chest tube functioned appropriately with no postoperative air leak, and serial postoperative chest radiographs demonstrated complete lung re‐expansion without residual pneumothorax (Figure 6). The patient remained hemodynamically stable throughout recovery, tolerated oral intake without nausea or dysphagia, maintained normal urinary output, and experienced no postdural puncture headache, urinary retention, neurological deficits, hoarseness, aspiration, respiratory compromise, wound complications, or other adverse events related to either the thoracic spinal anesthesia or the vagus nerve block. The chest tube was removed on postoperative day two after cessation of drainage and confirmation of complete lung expansion on chest radiography. The patient was discharged home in good general condition on postoperative day two with outpatient follow‐up arranged through the thoracic surgery clinic. At follow‐up, there was no clinical or radiographic evidence of recurrent pneumothorax, and the patient remained neurologically intact with complete recovery of normal swallowing and phonation.

## 3. Discussion

Thoracic surgery is conventionally performed under general anesthesia with endotracheal intubation and OLV, which provides excellent surgical exposure while allowing isolation of the operative lung. However, OLV and positive‐pressure ventilation are associated with well‐recognized physiological consequences, including ventilation–perfusion mismatch, ventilator‐induced lung injury, postoperative atelectasis, impaired mucociliary clearance, systemic inflammatory responses, and increased risk of postoperative pulmonary complications, particularly in elderly patients and those with limited cardiopulmonary reserve [1–3]. Consequently, interest has grown in NITS, which aims to preserve spontaneous ventilation while avoiding airway manipulation, neuromuscular blockade, and mechanical ventilation [2, 4–6].

Over the past decade, several observational studies and systematic reviews have demonstrated that NITS is feasible and safe in carefully selected patients undergoing procedures such as wedge resection, bullectomy, pleural biopsy, pleurodesis, sympathectomy, and even selected anatomical lung resections [4–8]. Reported benefits include lower postoperative pulmonary complication rates, reduced inflammatory response, shorter hospital stay, faster postoperative recovery, decreased need for intensive care admission, and improved preservation of respiratory physiology compared with conventional intubated thoracic surgery [4, 5, 7, 8]. Nevertheless, successful implementation requires careful patient selection, experienced thoracic surgeons and anesthesiologists, and immediate availability for conversion to general anesthesia should respiratory or surgical conditions deteriorate [2, 4, 7].

Several regional anesthetic techniques have been described for awake thoracic surgery, including thoracic epidural anesthesia, thoracic paravertebral block, erector spinae plane block, intercostal nerve blocks, and TSSA [3, 4, 9]. Among these techniques, TSSA offers several theoretical advantages, including rapid onset, reliable dense sensory blockade, minimal local anesthetic requirements, preservation of spontaneous ventilation, and limited sympathetic blockade when low‐dose local anesthetics are administered [3, 10]. Although thoracic epidural anesthesia has historically been considered the reference regional technique for thoracic surgery, TSSA may provide comparable surgical anesthesia while avoiding epidural catheter placement and reducing the risk of incomplete or patchy blockade [3, 9, 10].

Despite these potential advantages, thoracic spinal anesthesia remains relatively uncommon because of concerns regarding inadvertent spinal cord injury. However, anatomical magnetic resonance imaging studies have demonstrated the presence of a measurable posterior cerebrospinal fluid space within the mid‐thoracic region, providing a safety margin between the dura mater and the spinal cord [10, 11]. Clinical series involving experienced anesthesiologists have likewise reported low complication rates when thoracic spinal anesthesia is performed using meticulous technique, fine pencil‐point needles, and low doses of local anesthetic [3, 10, 11]. In the present case, a single midline puncture at the T5–T6 interspace using a 25‐gauge pencil‐point needle produced an effective sensory block extending approximately from T3 to T8 without paresthesia, neurological complications, significant hypotension, or bradycardia.

One of the major technical challenges during awake thoracic surgery is suppression of the cough reflex during manipulation of the lung and visceral pleura. Uncontrolled coughing may interfere with thoracoscopic visualization, compromise stapling accuracy, prolong operative time, and occasionally necessitate conversion to general anesthesia [4]. Various techniques have been described to suppress this reflex, including intrathoracic vagal infiltration performed by the surgeon, intravenous opioids, systemic sedation, stellate ganglion block, aerosolized local anesthetics, and ultrasound‐guided cervical vagus nerve block. Among these methods, ultrasound‐guided cervical vagus nerve block has gained increasing interest because it allows targeted suppression of the cough reflex while preserving spontaneous ventilation and minimizing systemic drug administration.

In our patient, ultrasound‐guided ipsilateral cervical vagus nerve block using 4 mL of 1% lidocaine provided complete suppression of the cough reflex throughout wedge resection and mechanical pleurodesis. The patient remained fully awake, maintained spontaneous ventilation, and required neither systemic sedative agents nor conversion to general anesthesia. Importantly, no complications attributable to vagus nerve blockade—including dysphagia, recurrent laryngeal nerve palsy, hoarseness, aspiration, airway compromise, or local anesthetic systemic toxicity—were observed during either the intraoperative or postoperative period. These findings are consistent with previously published reports demonstrating the effectiveness and safety of ultrasound‐guided vagus nerve blockade when performed by experienced operators.

Appropriate patient selection remains fundamental to the success of NITS and TSSA. Ideal candidates should be cooperative, able to tolerate the lateral decubitus position, capable of maintaining spontaneous ventilation, and free of contraindications to neuraxial anesthesia [2, 4]. Conversely, patients with severe obesity, anticipated difficult airway, inability to cooperate, uncontrolled coagulopathy, elevated intracranial pressure, or local infection at the puncture site may not be appropriate candidates for this approach [2, 4, 10]. Consequently, careful multidisciplinary assessment involving thoracic surgeons, pulmonologists, and anesthesiologists is essential before selecting awake thoracic surgery.

The present patient represented a particularly challenging anesthetic scenario because of advanced age, persistent spontaneous pneumothorax despite chest tube drainage and two autologous blood‐patch pleurodesis attempts, ongoing oxygen dependence, resolving pneumonia, cerebellar atrophy, parkinsonian tremor, and inability to undergo pulmonary function testing. Collectively, these factors substantially increased the anticipated perioperative risk associated with conventional general anesthesia and OLV. Following multidisciplinary evaluation, awake VATS under TSSA combined with cervical vagus nerve block was therefore considered the most appropriate anesthetic strategy.

Our patient experienced complete resolution of the persistent air leak, successful lung re‐expansion, uncomplicated postoperative recovery, and discharge on postoperative day two without neurological complications, postdural puncture headache, urinary retention, respiratory deterioration, dysphagia, hoarseness, aspiration, or need for postoperative mechanical ventilation. These favorable outcomes are consistent with previous reports suggesting that, in selected patients, awake thoracoscopic surgery under regional anesthesia may facilitate enhanced recovery while avoiding complications associated with airway instrumentation and positive‐pressure ventilation [4–8].

Nevertheless, several limitations should be acknowledged. This report describes a single patient and therefore cannot establish comparative efficacy or safety. Furthermore, direct comparison with conventional general anesthesia was not possible, and pulmonary function testing could not be performed because of the patient’s clinical condition. Larger prospective studies, multicenter registries, and randomized clinical trials are required to define optimal patient selection criteria, standardize anesthetic protocols, clarify the role of adjunctive vagus nerve block, and determine long‐term outcomes of TSSA in NITS.

## 4. Conclusion

This case demonstrates that awake VATS using TSSA combined with ultrasound‐guided cervical vagus nerve block was successfully performed in a carefully selected high‐risk patient with persistent spontaneous pneumothorax and significant pulmonary compromise. The technique provided satisfactory surgical conditions, effective suppression of the cough reflex, stable intraoperative hemodynamics, preservation of spontaneous ventilation, and an uncomplicated postoperative recovery without neurological or airway‐related complications.

Rather than suggesting replacement of conventional general anesthesia, this case supports the potential role of TSSA as an alternative anesthetic option in highly selected patients when a multidisciplinary team determines that avoidance of airway manipulation and OLV may be advantageous. Larger prospective studies are required to establish its safety profile, define appropriate patient selection criteria, and clarify its role within modern NITS.

The patient expressed satisfaction with the anesthetic and surgical experience. He reported acceptable postoperative pain control, was able to resume oral intake without difficulty, and experienced no neurological symptoms, dysphagia, or hoarseness following surgery. At follow‐up, he was pleased with the resolution of his respiratory symptoms and recovery.

## Funding

No funding was received for this manuscript.

## Conflicts of Interest

The authors declare no conflicts of interest.

## Supporting Information

Additional supporting information can be found online in the Supporting Information section.

## Supporting information


**Supporting Information** Timeline of Clinical Course.

## Data Availability

The data that support the findings of this study are available in the supporting material of this article.
