# Supplementary material for: Thoracic Segmental Spinal Anesthesia for Bullectomy in a Patient With Severe Pulmonary Compromise: A Case Report
Source: Case Rep Anesthesiol. 2026 Jul 19;2026:2418831. doi: 10.1155/cria/2418831 (PMC13381717; doi:10.1155/cria/2418831)
Supplement: Supplementary file 1 — Supporting Information Timeline of Clinical Course. [file CRIA-2026-2418831-s001.docx]

## **Timeline of Clinical Course**

### Month −1 (Initial illness)

- Patient developed intermittent productive cough and low-grade fever
- Diagnosed with community-acquired pneumonia
- Completed antibiotic therapy
- Infectious symptoms improved but respiratory status gradually worsened

### Days −10 to −5 (Progressive deterioration)

- Progressive hypoxemia despite clinical improvement from pneumonia
- Home pulse oximetry showed oxygen saturation decline to ~88%
- Increasing dyspnea developed

### Day 0 (Acute event / admission)

- Sudden severe dyspnea
- Oxygen saturation dropped to 72%
- Emergency presentation
- Needle decompression performed
- Left intercostal chest tube inserted after transfer

### Hospital course (Days 1–5 preoperative period)

- Persistent left pneumothorax despite chest tube drainage
- CT scan confirmed persistent air leak with mediastinal shift
- Resolving pneumonia noted radiologically
- Two autologous blood patch pleurodesis attempts performed
  - Partial improvement only
  - Persistent air leak remained
- Multidisciplinary decision for surgical management

Preoperative assessment period

- Severe respiratory compromise with oxygen dependence
- Pulmonary function testing not feasible
- High anesthetic risk for general anesthesia with one-lung ventilation
- Decision for awake VATS under thoracic segmental spinal anesthesia + vagus nerve block

### Day of surgery

- Thoracic segmental spinal anesthesia (T5–T6, 6 mg bupivacaine) performed
- Ultrasound-guided vagus nerve block performed
- Awake VATS bullectomy + mechanical pleurodesis completed
- No conversion to general anesthesia

### Postoperative period (Day 0–2)

- Stable respiratory and hemodynamic status
- No air leak postoperatively
- Lung fully re-expanded on imaging
- No neurological or airway complications
- Chest tube removed on postoperative day 2
- Discharged home on postoperative day 2

### Follow-up

- No recurrence of pneumothorax
- Normal swallowing and phonation
- Full neurological recovery
- Patient satisfied with outcome

| **Parameter** | **Finding** |
| --- | --- |
| Block level | T5-T6 |
| Spinal level | T5-6 |
| Needle | 25G pencil point |
| Bupivacaine | 6 mg |
| Vagus block | Left, 4 mL 1% lidocaine |
| Oxygen | Nasal cannula |
| Airway | None |
| Conversion | None |
| Hemodynamics | Stable |
| Complications | None |
